# The voiding efficiency in rat models with dopaminergic brain lesions induced through unilateral and bilateral intrastriatal injections

**DOI:** 10.1371/journal.pone.0243452

**Published:** 2020-12-03

**Authors:** Chellappan Praveen Rajneesh, Jian-Chiun Liou, Tsung-Hsun Hsieh, Jia-Hong Lin, Chih-Wei Peng

**Affiliations:** 1 School of Biomedical Engineering, College of Biomedical Engineering, Taipei Medical University, Taipei, Taiwan; 2 Department of Physical Therapy and Graduate Institute of Rehabilitation Science, College of Medicine, Chang Gung University, Taoyuan, Taiwan; 3 Neuroscience Research Center, Chang Gung Memorial Hospital, Linkou, Taiwan; 4 Research Center of Biomedical Device, Taipei Medical University, Taipei, Taiwan; Hokkaido Daigaku, JAPAN

## Abstract

Bladder dysfunction is a common phenomenon in Parkinson’s disease (PD) patients. A research attempt was made to analyze the voiding efficiency (VE) and bladder functions in rats with PD induced by unilateral or bilateral injections of 6-hydroxydopamine (6-OHDA) into the medial forebrain bundle. PD rats were divided into unilateral- and bilateral-injected groups and subjected to rotation and beam walking tests. Further, the experimental rats underwent cystometric measurements for analyses of bladder dysfunction and VE. Immunohistochemical analysis was performed to analyze the dopaminergic neuron depletion on the target area. Outcomes of the rotation and beam walking tests revealed the extent of parkinsonism in the experimental rats. Urodynamic observations denoted that rats with unilateral PD exhibited a significantly decreased VE (from 68.3±3.5% to 32.7±5.8%), while rats with bilateral PD displayed a much-reduced and substantially lower level of VE of 18.3±5.1% compared to the control value and to that of rats with unilateral PD. Rats with bilateral PD showed more-extensive behavioral deficits and urodynamic changes than did rats with unilateral PD. These significant changes in motor, behavioral, bladder function and VE were due to an extensive degeneration of dopaminergic neurons in the substantia nigra region on both sides of the brain. The obtained results were substantiated with appropriate immunohistochemical results.

## Introduction

Parkinson’s disease (PD) is a complex, progressive neurodegenerative disease (NGD), and it is the second most prevalent NGD predominantly due to depletion of dopaminergic (DA) neurons in the striatum (Str) and substantia nigra (SN) region of the brain [1]. As an NGD, notable symptoms are speech impairments, cognitive decline, gait deterioration, falls, gastrointestinal and genitourinary changes, and swallowing impairment [2]. In addition to these, literature evidence from Margolesky *et al*. [3] characteristically indicates that urinary dysfunction is common in PD patients.

The leading cause of PD is degeneration of DA neurons in the nigrostriatal and mesolimbic systems [4]. Complete elimination of DA neurons results in abnormal “burst” activity in the subthalamic nucleus (STN), which induces overreaction of basal ganglion output nuclei, ultimately leading to obstacles to voluntary movements [5]. For research purposes, typical rat models of PD are created by microinjecting the neurotoxic, highly oxidizable dopamine called 6-hydroxy dopamine (6-OHDA) into the medial forebrain bundle (MFB). This procedure can deplete >90% of catecholaminergic neurons, such as DA neurons in the SN and a >97% reduction in total striatal DA neurons. Eventually, a state analogous to the severely rigid akinetic terminal stage of human PD ensues [6].

Microinjection of 6-OHDA into one hemispheric side of the striatum of the nigrostriatal pathway is a common approach for PD model studies in rodents [7]. Few studies applied bilateral intrastriatal microinjections into the brain to induce PD. This is because 6-OHDA acts bilaterally on the SN, rats become hypokinetic and also aphagic and adipsic and are very difficult to maintain [8]. However, the degeneration of DA neurons in all PD patients commonly exists on bilateral sides rather than a unilateral side. Moreover, few studies have investigated urinary functions of a PD rodent model with bilateral 6-OHDA intrastriatal injections, and manifestations of unilateral and bilateral PD in rats have never been extensively compared. Therefore, in the present study, we compared motor functions and bladder function in rats with experimentally induced PD by unilateral and bilateral microinjections of 6-OHDA.

## Materials and methods

### Animals and the experimental design

**Fig 1:**
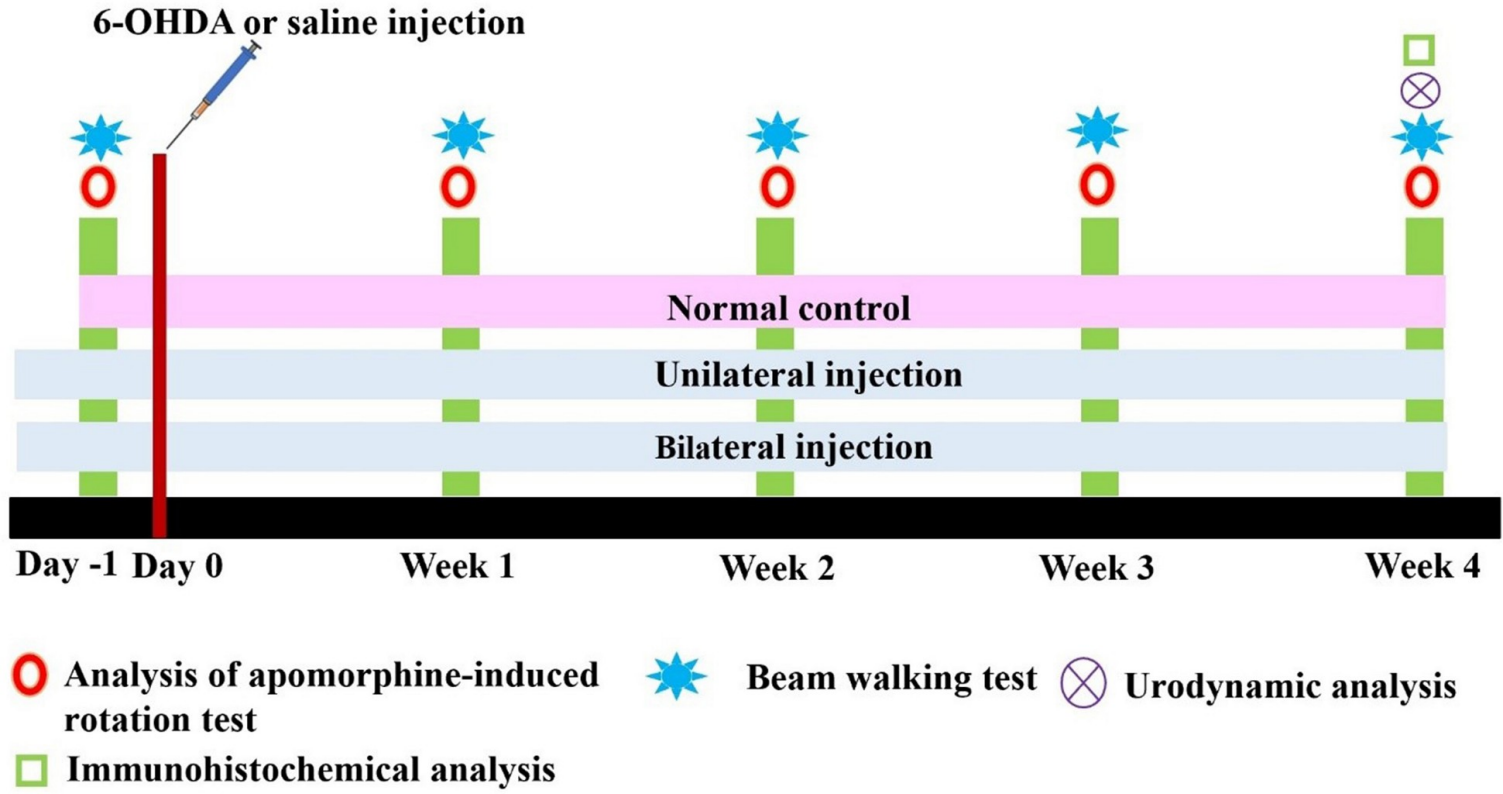
Study design and experimental schedule of the comparative analysis of rats with unilateral and bilateral Parkinson's disease (PD) along with normal control (NC) rats. The motor behavioral analysis was scheduled at 1 day before (day -1) and 1, 2, 3, and 4 weeks after the induction of unilateral and bilateral PD. The urodynamic and immunohistochemical analyses were scheduled for all groups at week 4, after the commencement of the motor behavioral analysis.

In total, 30 male Sprague-Dawley rats weighing 250~350 g were used in the study, and these were equally assigned to three groups, including normal control (NC), unilateral PD, and bilateral PD groups. Animal experiments were approved by the Institutional Animal Care and Use Committee (IACUC) of Taipei Medical University (LAC-2016-0408). Animals for the two PD groups were unilaterally or bilaterally given intrastriatal injections of 6-OHDA, and the NC group was given intrastriatal injections of saline. In all animals, motor behavioral tests were conducted 1 day before and 1~4 weeks after the intrastriatal injection intervention. Urodynamic measurements were performed on the same day after completing the last motor behavioral test (Fig 1). After the experiments, the rats were euthanized with an overdose of anesthetics drugs and the brains were collected for further immunohistochemical examination for the analysis of the DA neuron depletion.

### Induction of lesions in DA neurons by 6-OHDA

To induce PD in this animal model, rats were intraperitoneally anesthetized with tiletamine-zolazepam (50 mg/kg; Zoletil, Vibac, France) and positioned into a stereotactic apparatus (Stoelting, Wood Dale, IL, USA). The bregma line was carefully exposed by making an incision about 2 cm in the center of the head. For the microinjection, 4 μg/μl of 6-OHDA (dissolved in 0.1% ascorbic saline, Sigma Chemical, St. Louis, MO, USA) was injected intracranially into the MFB at an anteroposterior position of −0.4 mm from the bregma, lateral -1.6 mm to the midline, and ventral -7 mm from the skull surface [9]. This procedure was done on the left side of the brain using a unilateral injection with the aid of a 26-gauge 10-l Hamilton microsyringe (SETonic GmbH, Ilmenau, Germany) mounted vertically on the stereotactic frame, and similar procedures were conducted on both sides of the brain for the bilateral PD group. The syringe was sunk through a burr hole, and the toxin was infused at a rate of 0.5 μl/min with a syringe pump, for a total volume of 4 μl. The needle remained in the brain for no less than 5 min to preclude backfilling along the injection tract [10].

### Analysis of apomorphine-induced changes in the rotation test

Rats with unilateral DA lesions showed characteristic parkinsonian features, exhibiting typically slower general activities and also a tendency to turn toward the ipsilateral lesion side, but tended to turn toward the contralateral side after the apomorphine injection [11]. To determine whether we had successfully induced parkinsonian features in animals in the unilateral or bilateral PD groups, rotation tests after an apomorphine injection were conducted in all animals in this study. All animals received a subcutaneous injection in the neck with 0.05 mg/kg apomorphine hydrochloride (Sigma Chemical) dissolved in 1% ascorbic acid with 0.9% NaCl. A rat was placed on a transparent fiber testing bowl with a diameter of 30 cm and assessed over for 60 min [12]. 6-OHDA-lesioned rats which engaged in more than 100 rotations in 60 min were considered to have a nearly complete lesion, while the ones performing fewer than 100 rotations were classified as having an incomplete lesion [13, 14]. For identification, one of two differently colored stickers was pasted onto each rat's back. All data were observed and recorded in an image analyzer programmed and analyzed using MATLAB software.

### Beam-walking test

The beam-walking test was performed to investigate the akinesic phenomenon of PD rats. In this test, an animal had to cross a 2-m-long bar, which was placed between two blocks at over 9 cm above the basal surface. Each rat was placed on a block, and each forepaw was placed alternatively on a horizontal acrylic bar with a diameter of 0.7 cm. The forepaw nearest the camera was recorded as the starting point. The total time spent by each paw on the bar, i.e., the amount of time it took until the last step crossed the endpoint on the bar was recorded [10].

### Urodynamic analysis

After the motor behavioral tests, all rats were further subjected to cystometrographic (CMG) measurements in an anesthetized status (urethane, 1.25 g/kg body weight (BW), subcutaneous). An anesthetized animal was placed on a heated pad, with its urinary bladder and distal part of urethra opened wide through an abdominal incision. One end of a polyethylene (PE)-60 tube (with a 1.0-mm inside diameter and 1.5-mm outside diameter) was heated to form a collar-like structure to avoid dropping out of the bladder. It was then inserted into the bladder through a small incision at the apex of the bladder dome to measure the intravesical pressure (IVP), and the tube was secured with a purse-string suture. To record external urethral sphincter (EUS)-electromyographic (EMG) activity, two 50-μm epoxy-coated platinum-iridium wire electrodes (A-M Systems, Everett, WA, USA) were inserted at the tip of a 30-gauge needle and pinned into both sides of the EUS under direct visualization.

The abdominal wall was taped up with nylon sutures allowing the wire electrodes and bladder catheter to exit. The free end of the PE tube was attached by a 3-way stopcock to an infusion pump for filling with physiological saline and to a pressure transducer for monitoring the IVP. In addition, the wire electrodes were connected to a computer where CMG data were amplified and digitized. To analyze bladder activity, saline was introduced into the bladder via the PE-60 tube under the control of a saline injector at a rate of 0.12 mL/min. CMG and EUS-EMG parameters were both calculated using Acknowledge software (Biopac Systems, Goleta, CA, USA).

CMG variables were measured to quantify the effects of the unilateral and bilateral lesions on DA neurons by 6-OHDA including the micturition volume threshold (VT), considered the infused volume of saline adequate to induce the first voiding contraction; the contraction amplitude (CA), defined as the pressure determined during voiding; and the bladder contraction duration (CD) during voiding. ICI denotes the inter contraction interval which defines an intermediate time between two micturition phase. The VE is the ratio of the voided volume (VV) to the VT. The VV was calculated as the VT subtracted from the residual volume (RV) of saline introverted through the intravesical catheter after the final voiding contraction [15].

When voiding, EUS-EMG activity was observed as a result of the burst activity of the EUS. At the time of voiding, observations of EUS-EMG depicted active periods (APs) and silent periods (SPs) situated inside the burst period (BP). The BP was defined as the period between the starting point and the terminal end of voiding. Alternatively, the transformation of tonic EMG into a burst discharge was completed when the EMG was converted to tonic EMG. The SP was defined as a quiet period flanked by two high-frequency spikes, which was further well defined as a static period sandwiched between the transformation point of high-frequency spikes into low-frequency waves and vice versa. APs are high-frequency spikes that are separated by an idle period, and an AP can also be demarcated as the period flanked by low-frequency waves transformed to high-frequency spikes and contrariwise [15].

#### Immunohistochemical analysis

For the analysis of DA depletion in the PD rats, immunohistochemical analysis was performed. Three rats (*n* = 3) from each group were selected for the study. After the urodynamic examination, the animals were sacrificed, and the brains were removed sacrificed for tyrosine hydroxylase (TH) staining. The experimental procedure for immunohistochemical analysis was performed as previously reported [10]. The brain sections were incubated with normal goat serum (Histofine Nichirei Co., Ltd. Tokyo, Japan), subsequently with rabbit anti-TH (Chemocon Co Ltd, Tokyo, Japan) and biotinylated goat anti-rabbit IgG secondary antiserum (Histofine Nichirei Co., Ltd. Tokyo, Japan) was applied. Later, the sections were incubated with peroxidase-conjugated streptavidin (Histofine Nichirei Co., Ltd. Japan). During incubation, sections were rinsed and were allowed to react with 3,3´-diaminobenzidine as a chromogen. The sections were mounted on gelatin-chromalum coated slides and later the slides were dehydrated and coverslipped.

### Statistical analysis

All data are presented as the mean±standard deviation (SD). A one-way analysis of variance (ANOVA) was used for overall comparisons among the three animal groups. Differences between groups were investigated by a *post-hoc* analysis using Tukey's honest significant difference method, and *p* values of <0.05 were accepted as the significance level. All statistical analyses were performed using the GraphPad Prism 6 statistical package (GraphPad Software, San Diego, CA, USA).

## Results

### Rotation test

**Fig 2:**
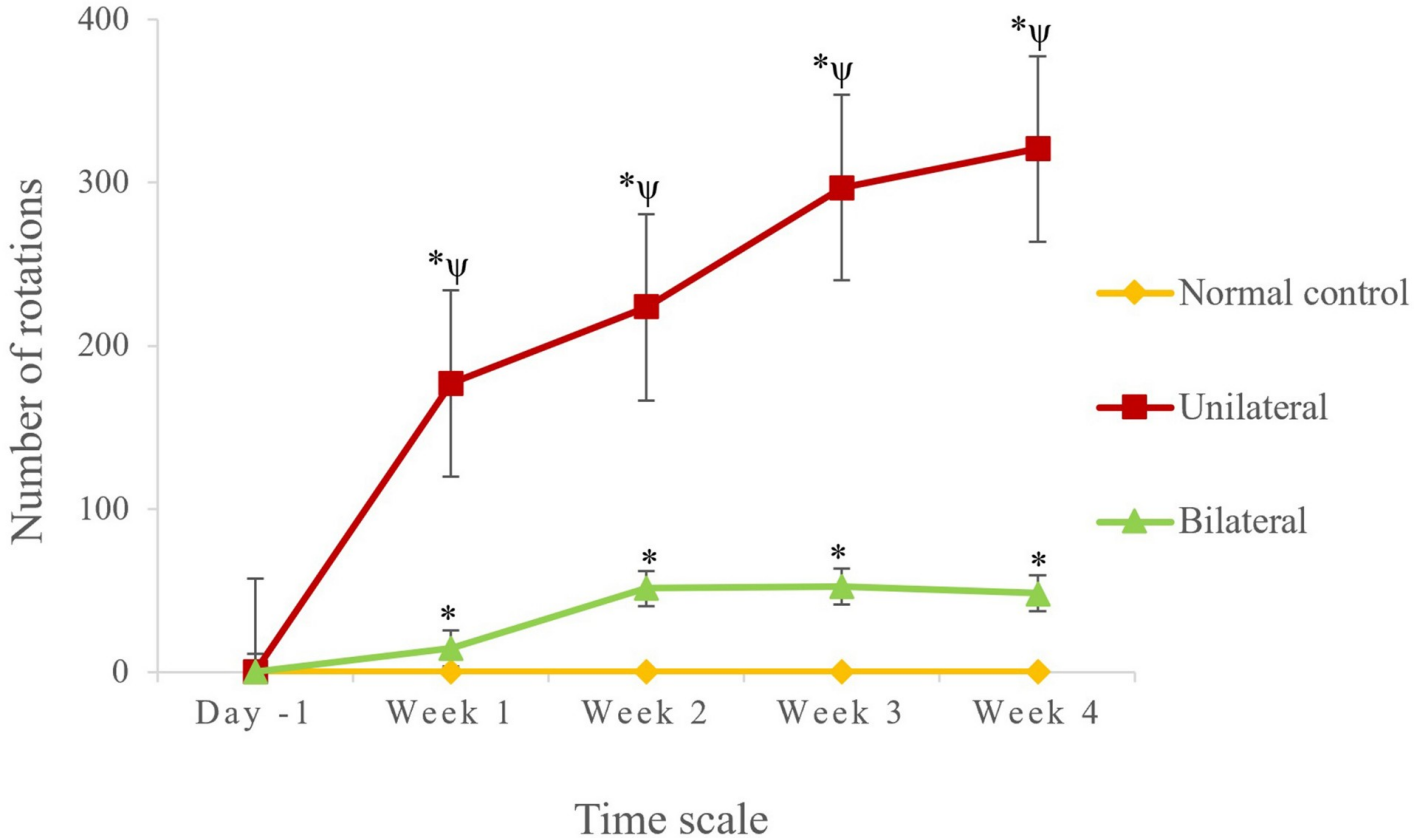
Results of a comparative analysis of the rotation test among normal control (NC) rats, and with rats unilateral and bilateral Parkinson's disease (PD). *, Indicates a significant difference (*p*<0.05) compared to the NC group. ^ψ^, Indicates a significant difference (*p*<0.05) in rat with unilateral PD compared to the corresponding time point of rats with bilateral PD.

Fig 2 depicts changes in rotation behavior among rats with unilateral and bilateral PD and NC rats from 1 day before and 1~4 weeks after induction of lesions in DA neurons. Results of day -1 (1 day before the injection) showed no significant difference among the three groups. However, both the unilateral and bilateral PD groups exhibited a significant and increasing level of rotations at all-time points from weeks 1 to 4 compared to the NC value at the corresponding time point (*p*<0.05). Interestingly, the bilateral PD group exhibited a significantly lower level of rotations at all-time points compared to the unilateral PD values.

### Beam-walking test

**Fig 3:**
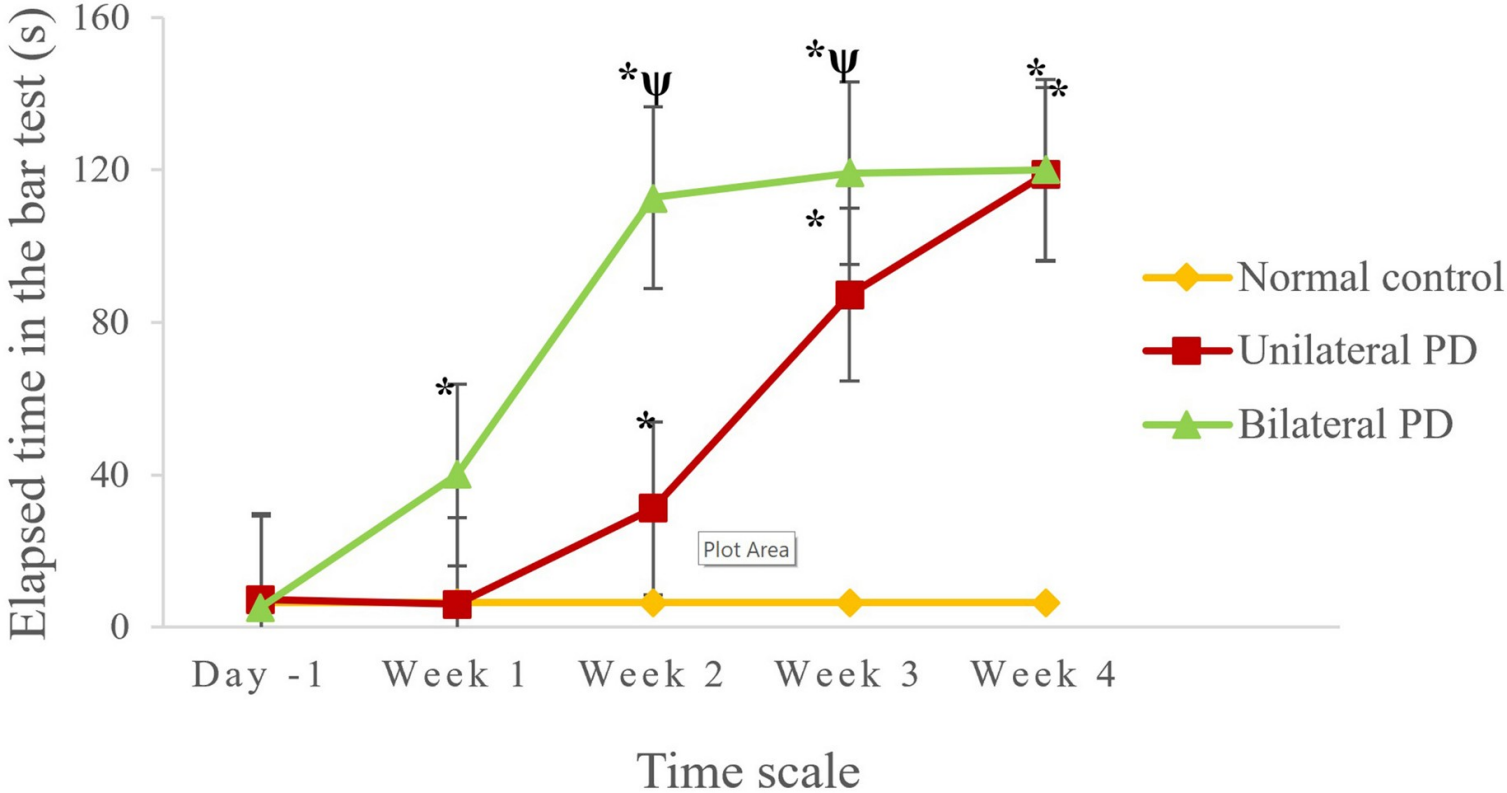
Results of a comparative analysis of the beam walking test among normal control (NC) rats, and rats with unilateral and bilateral Parkinson's disease (PD). *, Indicates a significant difference (*p*<0.05) compared to the NC group in rats with both unilateral and bilateral PD. ^ψ^, Indicates a significant difference (*p*<0.05) in rats with bilateral PD compared to the corresponding time point in rats with unilateral PD.

Fig 3 shows results of the beam walking test in the unilateral and bilateral PD groups from 1 day before to 1~4 weeks after the 6-OHDA injection. The two experimental groups before the PD induction displayed no differences in walking patterns or time differences compared to NC rats. Later, unilateral PD rats at 1 week after PD induction still showed a normal pattern of walking, and no significant difference was observed compared to the NC value. However, the unilateral PD rats displayed languid and typical parkinsonian movements with a significant time difference compared to NC rats in weeks 2 to 4. Notably, bilateral PD rats exhibited typical parkinsonian movements and significantly increased beam-walking times earlier from week 1 which lasted to week 4 compared to the unilateral PD and NC rats. In detail, all bilateral PD rats were unable to walk, and they stayed a maximum time on the bar. In addition, six of ten rats fell off the bar.

### CMG activity

**Fig 4:**
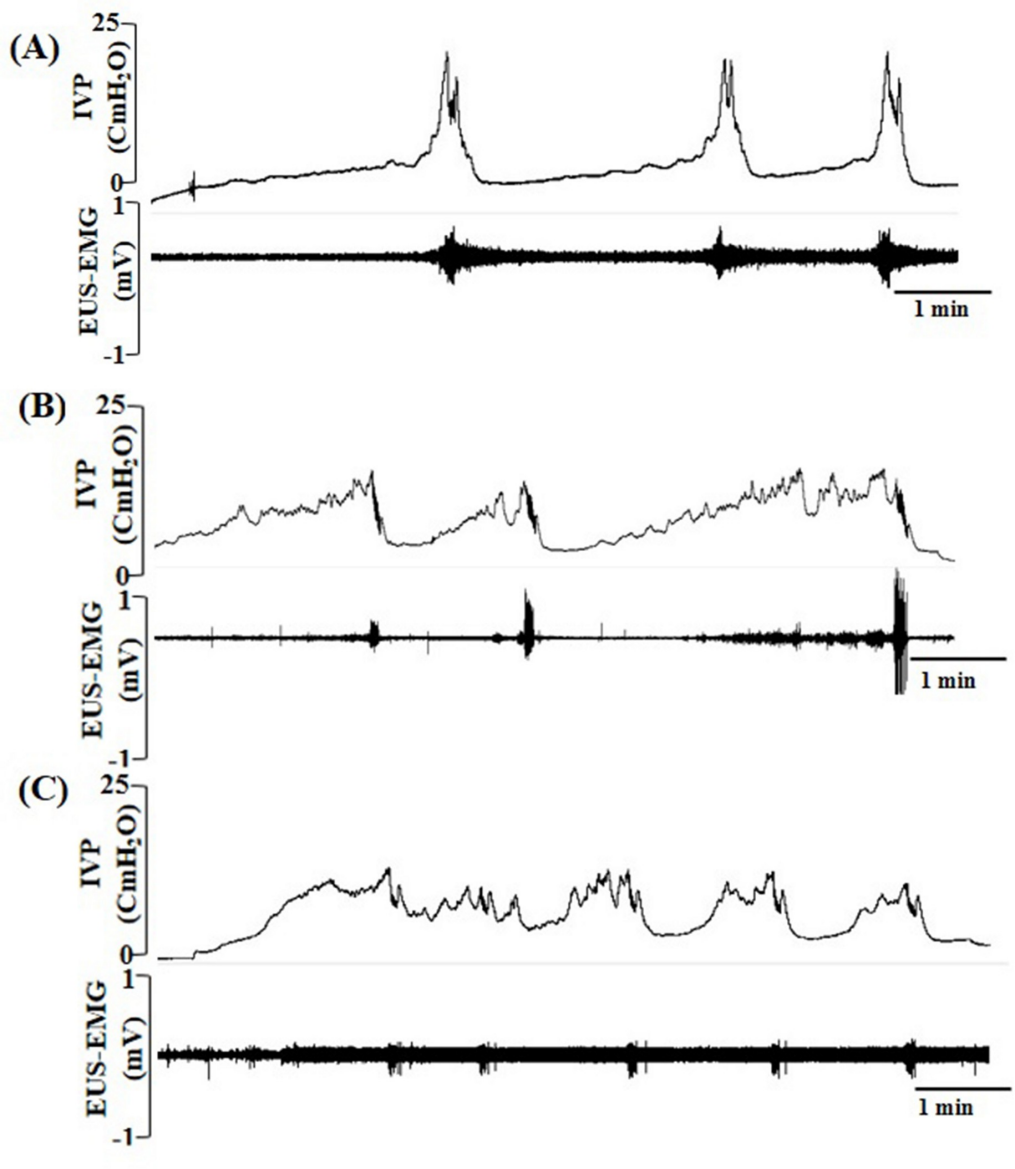
Typical pattern of intravesical pressure (A, top trace) and external urethral sphincter electromyographic (EUS-EMG) activity (bottom trace) recorded during a continuous transvesical infusion cystometrographic (CMG) measurement in anesthetized normal control (NC) rats (A), and rats with unilateral (B) and bilateral Parkinson's disease (PD) (C).

Comparative effects of unilateral and bilateral 6-OHDA injections on CMG parameters are summarized in Table 1. During CMG measurements, the average VT was slightly decreased in rats with unilateral and bilateral PD (by about 0.59~0.58 ml) compared to that of the NC group (about 0.64 ml) as shown in Fig 4, but these differences did not reach a significant level. Average CA values in rats with unilateral and bilateral PD were significantly lower compared to NC rats, and CA values of rats with bilateral PD significantly differed from those of rats with unilateral PD. Also, the CD was significantly higher by approximately 10%~15% above the control value in rats with unilateral and bilateral PD. ICI values were remarkably reduced in rats with unilateral (97.25±10.99 s, *p*<0.05) and bilateral PD (54.71±8.54 s, *p*<0.05) compared to values of NC rats (140.36±4.64 s) as depicted in Fig 4; differences among the three groups were statistically significant.

**Table 1:** Comparative cystometric measurements among normal control (NC) rats, and rats with unilateral and bilateral Parkinson's disease (PD).

	VT (ml)	CA (cmH_2_O)	CD (s)	ICI (s)	RV (ml)	VV (ml)	VE %
**NC rats**	0.64±0.02	42.37±3.86	20.27±3.03	140.36±4.64	0.21±0.03	0.43±0.01	68.3 ± 3.5%
**Unilateral PD rats**	0.59±0.05	26.28±8.87*	31.04±6.38*	97.25±10.99*	0.37±0.05*	0.19±0.03*	32.7±5.8%*
**Bilateral PD rats**	0.58±0.05	21.50±7.13*^ψ^	34.25±7.10*^ψ^	54.71±8.54*^ψ^	0.47±0.07*^ψ^	0.10±0.03*^ψ^	18.3±5.1%*^ψ^

*, Indicates a significant difference compared to the NC group.

^**ψ**^, Indicates a significant difference compared to animals unilaterally injected with 6-hydroxydopamine. Significance levels are marked at *p*<0.05. Values are the mean±SD; *n* = 10 in all groups.

VT, volume threshold; CA, contraction amplitude; CD, contraction duration; ICI, inter contraction interval; RV, residual volume; VV, voided volume; VE, voiding efficiency.

RV values were significantly higher in rats with unilateral (approximately 1.5-fold of the control) and bilateral PD (approximately 2-fold of the control) compared to values (0.21 ml) in NC rats. Moreover, the RV in rats with bilateral PD was significantly larger than that in rats with unilateral PD. The average VV of rats with bilateral PD (*p*<0.05) was significantly lower compared to values in control rats and rats with unilateral PD (*p*<0.05). The average VE of rats with unilateral PD was remarkably reduced (32.7±5.8%) to almost half of the value of NC rats (68.3±3.5%). The average value of rats with bilateral PD (18.3±5.1%, *p*<0.05) was significantly lower than and almost one-third those of NC rats and rats with unilateral PD.

### EUS-EMG activity

Table 2 summarizes EUS-EMG values among the NC, unilateral PD, and bilateral PD groups. The BP value of rats with unilateral PD (2.94±0.08 s) was significantly reduced compared to that of NC rats 3.63±0.41 s. Likewise, the BP of rats with bilateral PD was further significantly reduced compared to those of rats with unilateral PD and NC rats and exhibited a value of 1.99±0.07 s (*p*<0.05). The AP of rats with unilateral PD (89.31±0.76 ms) and bilateral PD (90.25±1.10 ms) displayed no significant difference compared to that of NC rats (91.46±2.84 ms). The SP of rats with unilateral PD at 110.94±1.29 ms was significantly lower compared to that of NC rats at 168.96±2.69 ms. Notably, rats with bilateral PD exhibited an extremely and remarkably reduced SP value of 59.99±1.07 ms (*p*<0.05), which significantly differed from those of rats with unilateral PD and NC rats.

**Table 2:** Comparative analysis of external urethral sphincter electromyographic (EUS-EMG) activity in normal control (NC) rats, and rats with unilateral and bilateral Parkinson's disease (PD).

	Burst period	Active period	Silent period
	(s)	(ms)	(ms)
**NC rats**	3.63±0.41	91.46±2.84	168.96±2.69
**Unilateral PD rats**	2.94±0.08*	89.31±0.76	110.94±1.29*
**Bilateral PD rats**	1.99±0.07*^ψ^	90.25±1.10	59.99±1.07*^ψ^

Values are the mean±SD; *n* = 10 for all groups. The frequency of the burst discharge is represented as the ratio between the number of silent periods and its burst period.

*, Indicates a statistically significant difference from NC rats.

^ψ^, Indicates a statistically significant difference from rats with a unilateral 6-hydroxydopamine injection. *p*<0.05.

### Immunohistochemical analysis

**Fig 5:**
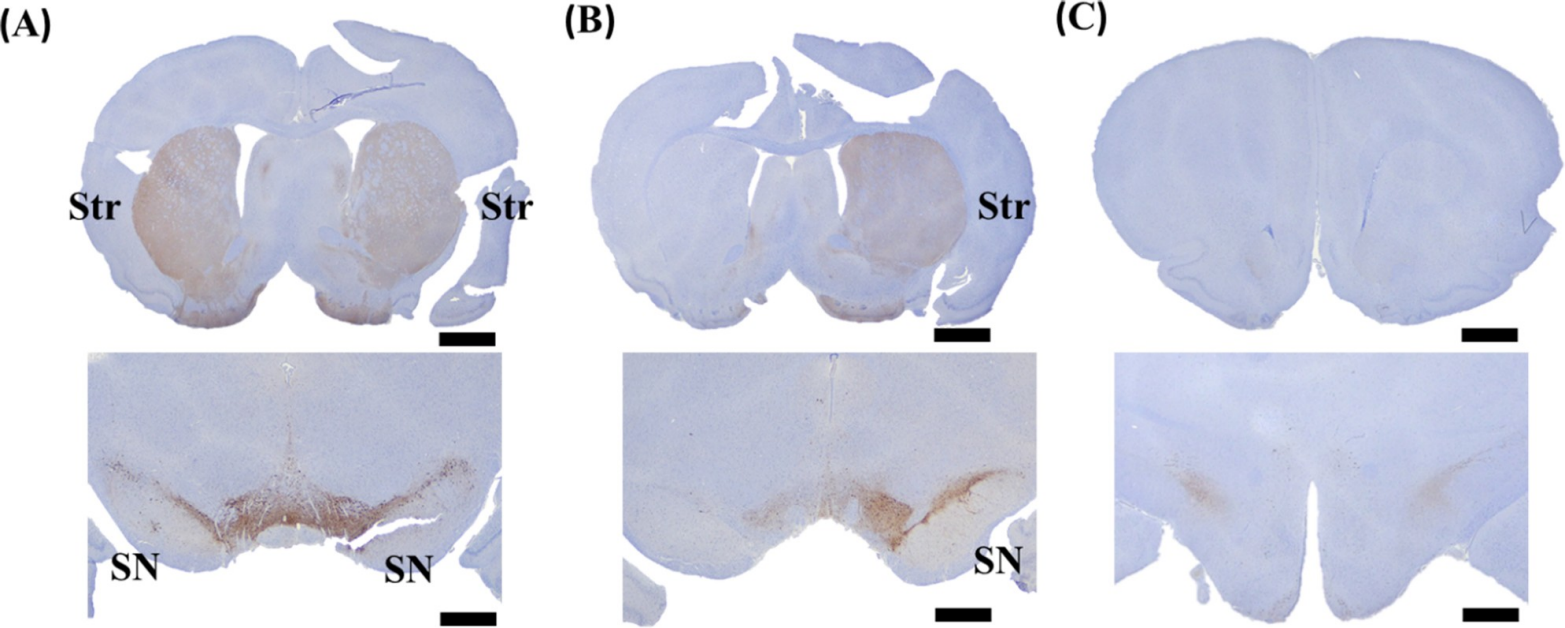
Representative micrographs of tyrosine hydroxylase (TH) staining sections of (A) normal control (NC) rats with intact DA neurons at bilateral sides of striatum (Str) and SN regions (brown-color regions), (B) one side of intact DA neurons in unilateral PD rats, and (C) nearly both side depletion of DA neurons in bilateral PD rats. Scale bar, 2 mm.

Tyrosine hydroxylase-staining immunoreactivity in the striatum and substantia nigra (SN) regions was examined in each group of rats (*n* = 3 for each group) after 4-week of 6-OHDA lesion (Fig 5). The immunohistochemical results indicated a remarkable decrease in the catecholamine positive cells situated in the striatum and SN regions on the lesioned side in unilateral PD rats. Likewise, the significant depletion of the catecholamine positive cells was observed on both sides of the striatum and SN regions in bilateral PD rats compared with the NC rats.

## Discussion

Studies related to PD generally focused on behavioral and cognitive functions [4, 6]. In addition, urodynamic studies commonly used a unilateral PD model. The present study is a pioneer attempt to synchronously evaluate motor behavioral, bladder functions in rats with unilateral and bilateral PD. In this study, compared to the condition preceding the injection, rats administered a unilateral or bilateral injection showed changes not only in motor behavioral functions but also in bladder function and VE. Rats receiving an intrastriatal infusion of 6-OHDA are often used as an animal model of PD, and the use of a dopamine agonist such as apomorphine is extremely effective in measuring the rotational asymmetry in 6-OHDA-administered rats. It was reported that an imbalanced condition of DA receptors gives rise to a tendency for the rats to rotate [16]. Rats with unilateral PD typically exhibit a pattern of rotation data that are stable and have an increasing index of turnovers at all-time points compared to NC rats. This condition is due to an intrastriatal injection of 6-OHDA that produces extensive degeneration of DA neurons in the SN region through deteriorating axonal transport at dopamine striatal terminals [12, 17], as evidenced in the immunohistochemical results (Fig 5). These associated neurocellular modifications result in characteristic motor and urinary dysfunctions, which show some similarity to those observed in PD.

Interestingly, the significantly reduced number of rotations in rats with bilateral PD indicates the progressive loss of DA neurons on both sides of the striatum. Typically, rats with unilateral PD tend to rotate towards the side opposite the lesion, due to dominance of the DA receptor activity in the striatum on one side, which forces the animal to rotate to the other side [12]. Degeneration of DA nerve terminals encompasses several modifications in the neuronal environment on both sides of the corpus striatum region, which might also include a loss of symmetry when making voluntary movements. Thus, rats with bilateral PD were unable to effectively perform like rats with unilateral PD. It was proven that after DA neurons are destroyed in adult rats, there is a decrease in cerebral activation necessary for voluntary behaviors [18]. Our results also displayed that apomorphine-induced rotational alterations were well correlated with the progressive loss of dopamine neurons at all-time points.

Due to the conditions described above, we observed that rats bilaterally injected with 6-OHDA revealed a very long latency or total time on the beam compared to NC rats. This condition was due to the bilateral injection of 6-OHDA into the MFB that might have resulted in massive depletion of dopamine to the striatum [19]. Besides, there is a chance for postsynaptic alterations resulting from DA terminal destruction, thus resulting in several behavioral defects that mimic aspects of PD [20]. From a behavioral analysis, the present study indicated that rats bilaterally injected with 6-OHDA displayed several behavioral glitches compared to unilateral PD rats. This condition was mainly due to the massive depletion of DA neurons in the medial forebrain region.

Clinically, patients with both storage and voiding disorders are the most common findings in PD [21]. The PD patients mainly manifest urinary symptoms, including detrusor hyperreflexia during the filling phase and impaired contraction (a weak detrusor) during the voiding phase, which was defined as detrusor hyperactivity with impaired contractility (DHIC). According to statistical data [22–24], around 16%–54% of PD patients have the bladder storage problems such as urinary urgency and frequency, which may be severe, and urge incontinence particularly if poor mobility compounds their bladder disorder. Furthermore, a clinical study reveals that PD patients with urinary symptoms exhibited detrusor hyperreflexia during the cystometric filling phase with 64% and voiding-phase disorders (weak detrusor contractility) a level of 28% [21]. These clinical symptoms are consistent with the CMG results of the current study, as evidenced by detecting a decrease in the VT, CA, ICI, VE values, and an increase in the RV values in both unilateral and bilateral PD rats (Table 1). The storage and voiding dysfunctions in the bilateral PD rats were more prominent than those in the unilateral PD rats. The decrease in VT and ICI reflects a storage disorder in bladder capacity and detrusor hyperreflexia. Besides, compared to NC rats, the typical intravesical pressure patterns during a continuous CMG measurement in both PD groups exhibited pre-voiding bladder contractions (or no voiding contractions) during filling phase, which again displayed the symptom of detrusor hyperreflexia in our PD models (Fig 4).

The voiding disorder in PD patients was a subclinical symptom compared to the primary storage disorder, which may manifest in early and untreated PD patients [21]. However, few PD patients may have severe voiding disorders characterized by a significant increase in post-voiding residual urine. In relation to this, a clinical study which indicates that around 10% of PD patients with urinary symptoms have a ≥ 50% post-voiding residual urine [21, 25]. The results of the present study reveal that both the unilateral and bilateral PD groups had several voiding abnormalities, including decreases in CA, VV, and VE, whereas RV was increased. The decrease in CA represented weak detrusor contractility during bladder voiding contractions. Hence, the condition mentioned above might reflect the reduction in the urethral pulsatile force to expel urine. Thus, the detection of voiding inefficiency was not unexpected in both PD groups, as evidenced by a significant decrease in VV and VE and an increase in RV (i.e., residual urine). These findings were consistent with previous studies indicating that PD patients might manifest weak detrusor contractility during the voiding phase [25, 26] and an increased post-voiding residual urine [21]. Although only a small proportion of PD patients might exhibit the occurrence of severe voiding dysfunctions clinically, subsequently, the present observations of the voiding dysfunctions in PD rats might partially relevant to the clinical conditions. However, it is unclear whether our results apply to humans since the lower urinary tract’s neurophysiological control may differ from that in rats. Thus, the mechanisms of storage and voiding disorders in PD populations should be investigated in additional animal and clinical studies.

Several recent studies further indicated that the urinary symptoms are highly correlated with the severity and duration of motor disorders [26–28] in that a previous study specified that post-voiding residual urine volume was related to motor disorders [25]. There was an inverse relation between post-voiding residual urine volume and detrusor contractility (e.g., detrusor power) [26]. In the present study, the observed results displayed that the motor and voiding dysfunctions in the bilateral PD rats had a worse outcome than those in the unilateral PD rats. The bilateral PD rats exhibited a significant reduction in the walking speed and bladder contractility during voiding contraction compared to other groups. Thus, the present animal findings were supported by these clinical data. However, it remains unclear about the physiological mechanisms of the correlation between urinary symptoms and motor disorders. Consequently, in-depth studies must be warranted in this area for a better understanding.

The EUS-EMG recordings after the 6-OHDA injection in rats with unilateral and bilateral PD revealed burst activity, which indicates the rhythmic opening and closing of the outlet to produce a pulsatile flow of urine. For the EUS-EMG analysis (Table 2), the BP of rats with unilateral and bilateral PD invariably decreased compared to NC rats. This condition was mainly due to a reduction in bladder capacity. It was documented that the degeneration of DA neurons in the nigrostriatal pathway causes bladder hyperactivity through a reduced bladder capacity [29]. Moreover, it was also further documented that the microinjection of dopamine over the PMC has the ability to reduce bladder capacity [30]. In addition, the exact mechanism underlying bladder dysfunction in PD has still not been fully explained.

The SP of the EUS-EMG, which represents the relaxation and opening of the outlet, is indispensable for accomplishing efficient voiding [31]. In the present study, decreased values of SP indicated that the urethra was open for a shorter period during voiding, which could contribute to the decreased VE in rats with unilateral and bilateral PD. Hence, the VE of rats with unilateral PD was reduced compared to that of NC rats, while in rats with bilateral PD, the VE was drastically reduced compared to rats with unilateral PD and NC rats. Voiding is facilitated by reflex mechanisms that are well controlled in the brain. In the brain, the PMC facilitates storage and micturition according to signals it receives from the periaqueductal gray (PAG). Excitation of the PMC triggers descending pathways that initiate urethral relaxation, and within a few seconds, the sacral parasympathetic outflow is activated [32].

Triggering by the PMC results in contraction of the bladder, and the simultaneous increased intravesical pressure aids the flow of urine [33]. It was reported that drugs injected into the PMC could alter the setpoint level (i.e., the bladder volume that triggers micturition) for activating the switch without altering the extent of the voiding reflex [34]. The SP is mainly related to suppression of reflexes [31]. Hence, as substantiated above, there is a possibility that drugs injected into the PMC might have altered SP values in the present study. Hence, according to previous evidence and the affirmative results obtained in the present study, depletion of DA neurons giving rise to poor VE in the PD rat model is strongly supported. Additionally, a comparative analysis revealed that rats with bilateral PD exhibited a deplorable VE condition compared to rats with unilateral PD due to the massive depletion of DA neurons on both sides over the striatal region.

Additionally, the immunohistochemical evidence of unilateral PD rats displayed a significant decrease in the content of DA neurons on the confined area of the 6-OHDA injected side, whereas the bilateral PD rats exhibited DA neuronal loss on both sides. The previous reports in MFB-lesioned rats stated that the determination of DA suggested complete denervation of striatum after 3 weeks [35], which supported the results of the current study. The obtained results from the 4-week unilateral and bilateral PD model rats also exhibited an impaired motor behavioral condition on account of the apomorphine-induced rotation and the beam walking test, which reflected the neuronal loss on respective sides. It is also noteworthy that the loss of dopaminergic neurons based on 6-OHDA has a tendency to be dichotomy in rodents and the loss of neurons in PD animal models should be higher than in PD patients. Therefore, it is not clear whether the present motor behavioral and urodynamic results will translate to humans, where the depletion of DA neurons in histopathological progress may differ from that in the rat.

## Conclusions

In conclusion, our results indicated that unilateral and bilateral intrastriatal 6-OHDA injections in rats produced significant differences in behavioral, urological, and immunohistochemical defects induced by 6-OHDA. These data support the urological changes, particularly in the voiding efficiency, of parkinsonian rats. Bilateral terminal 6-OHDA lesions of the striatum led to more-extensive behavioral deficits and urodynamic changes, whereas unilateral lesions produced less damage than bilateral lesions. Furthermore, it was also clearly enumerated that functional impairments occurred with moderate and complete depletion of DA neurons and the nigrostriatal pathways. Further, these results may yield new insights into basal ganglia and DA neurons. Moreover, these findings may also provide an expedient experimental model for neuroprotective studies and rehabilitation approaches, particularly in restoring bladder function in PD patients.
